# B-Cell Activating Factor as a Cancer Biomarker and Its Implications in Cancer-Related Cachexia

**DOI:** 10.1155/2015/792187

**Published:** 2015-08-03

**Authors:** Michal Rihacek, Julie Bienertova-Vasku, Dalibor Valik, Jaroslav Sterba, Katerina Pilatova, Lenka Zdrazilova-Dubska

**Affiliations:** ^1^Department of Pediatric Oncology, University Hospital and Faculty of Medicine, Masaryk University, Cernopolni 9, 613 00 Brno, Czech Republic; ^2^Regional Centre for Applied Molecular Oncology, Masaryk Memorial Cancer Institute, Zluty Kopec 7, 656 53 Brno, Czech Republic; ^3^Department of Pathological Physiology, Faculty of Medicine, Masaryk University, Kamenice 5, 625 00 Brno, Czech Republic; ^4^Advanced Cell Immunotherapy Unit, Department of Pharmacology, Faculty of Medicine, Masaryk University, Kamenice 5, 625 00 Brno, Czech Republic

## Abstract

B-cell activating factor (BAFF) is a cytokine and adipokine of the TNF ligand superfamily. The main biological function of BAFF in maintaining the maturation of B-cells to plasma cells has recently made it a target of the first FDA-approved selective BAFF antibody, belimumab, for the therapy of systemic lupus erythematosus. Concomitantly, the role of BAFF in cancer has been a subject of research since its discovery. Here we review BAFF as a biomarker of malignant disease activity and prognostic factor in B-cell derived malignancies such as multiple myeloma. Moreover, anti-BAFF therapy seems to be a promising approach in treatment of B-cell derived leukemias/lymphomas. In nonhematologic solid tumors, BAFF may contribute to cancer progression by mechanisms both dependent on and independent of BAFF's proinflammatory role. We also describe ongoing research into the pathophysiological link between BAFF and cancer-related cachexia. BAFF has been shown to contribute to inflammation and insulin resistance which are known to worsen cancer cachexia syndrome. Taking all the above together, BAFF is emerging as a biomarker of several malignancies and a possible hallmark of cancer cachexia.

## 1. The BAFF/BAFF-Receptor System Is Essential for B-Cell and Plasma Cell Development and Function

B-cell activating factor (BAFF, BLyS, TNFSF13B, TALL-1, and CD257) is a 285-amino-acid type II transmembrane protein that belongs to the superfamily of 19 known TNF ligands [1, 2]. Since its discovery, BAFF has been confirmed as a necessary element in B-cell proliferation and as a specific immunity response enhancer [3]. BAFF deficiency leads to almost complete loss of follicular and marginal zone B-cell production in murine secondary lymphoid organs [4]. BAFF neutralization by soluble receptor decoys blocks the Th1 to Th2 transition, thereby leading to inhibition of antigen-specific antibody production [4, 5]. BAFF also mediates immunoglobulin isotype switching in B-cells [6]. BAFF signaling is potentiated by BCR ligation [7] and enhances survival in B-cells via activation of NF-*κ*B pathway.

Three receptors from the 29-member TNF receptor superfamily are now confirmed to interact with BAFF: BAFF-R, TACI, and BCMA [8] (Table 1). BAFF-R seems to be the most important receptor for BAFF, with a critical role in regulating B-cell survival [9]. Mice with a naturally occurring mutation in the BAFF-R locus (A/WySnJ mice) have a qualitatively similar phenotype to mice with BAFF deficiency, suggesting a unique role of BAFF-R in B-cell development that cannot be compensated by the two other BAFF receptors, TACI and BCMA [9, 10].

Unlike BAFF-R, TACI binds two ligands from the TNF superfamily: BAFF and APRIL [11]. The role of TACI in BAFF signaling is complex, as it induces both activation and inhibition of the NF-*κ*B pathway. When ligated by TACI, BAFF has been shown to be a negative regulator of B-cell expansion. TACI^−/−^ mice show B-cell hyperplasia and elevated levels of circulating antibodies, resulting in fatal autoimmune glomerulonephritis and splenomegaly [11, 12].

The primary ligand of BCMA is APRIL, although BAFF also binds to this receptor, albeit with low affinity [8]. A significant role for BCMA was determined in multiple myeloma. BCMA ligation provides survival signals for abnormal plasma cells to evade apoptosis [30, 32]. Notably, all three BAFF receptors activate NF-*κ*B pathways via TRAF signaling molecules [8, 33, 34].

## 2. Cytokine and Adipokine BAFF Is Expressed Ubiquitously

BAFF is expressed primarily as a membrane bound protein but is also extensively cleaved to a soluble form [35, 36]. Soluble BAFF levels in blood are related closely to the number of circulating B-cells and the amount of BAFF receptors available for cleavage. The normal levels of soluble BAFF in healthy adults range from 0.3 to 2.25 ng/mL in peripheral blood. The cord blood of newborns contains significantly higher concentrations of BAFF, ranging from 0.6 to 4.5 ng/mL [37].

The homotrimeric soluble form of BAFF activates BAFF-R. Homotrimeric BAFF can undergo oligomerization that is required for activation of TACI [11]. The expression of BAFF is not related to a single tissue or a specific group of cells. BAFF is expressed on the surface of human myeloid lineage cells (monocytes), primary and secondary lymphoid organs (spleen, bone marrow, and lymph nodes), and various tissues that do not possess primary immune functions (e.g., low expression levels in heart and pancreas) [38]. Moreover, expression of BAFF and its receptors was confirmed in human adipose tissue cultures [17]. In a mouse model, BAFF expression was upregulated during adipocyte differentiation and under proinflammatory conditions (treatment with TNF-*α*) [39]. BAFF also negatively affects insulin sensitivity in murine visceral adipose tissue [40]. In light of these findings, BAFF, being a cytokine and member of the adipokine family, is considered an important player in many pathophysiological conditions, including inflammation, autoimmune disorders, primary immunodeficiencies [39, 41, 42], obesity, and diabetes [40, 43, 44]. Along with its connection to the apoptosis regulating NF-*κ*B signaling pathway, the role of the BAFF ligand/receptor system in malignant diseases is steadily being elucidated [31, 45].

## 3. Expression of BAFF Is Regulated by Interferon and Estrogen Levels

Gamma interferon activation site (GAS) element was described in the promoter region of* BAFF* gene in human intestinal epithelial cells leading to IFN-*γ*-induced expression of BAFF via activation of JAK/STAT signaling [46]. This molecular mechanism of BAFF regulation was supported by correlation of IFN-*γ* and BAFF levels observed in various human immune system-related cells under physiological and malignant conditions [47–49]. Moreover, therapeutic IFN-*β* administration also increases BAFF levels* in vivo* [50, 51]. Hence, BAFF can be considered as a molecule that connects innate and specific immunity through its response to IFN-*γ* and IFN-*β* and its subsequent activation of B-cells.

Interestingly, BAFF expression is enhanced in the presence of elevated estrogen levels in mice with systemic lupus erythematosus [52] and estrogen-induced B-cell activation in lupus mice is blocked by the antiestrogenic activity of tamoxifen. Thus, estrogen-induced BAFF upregulation may contribute to a higher incidence of autoimmune disorders in females [53].

## 4. BAFF Antiapoptotic and Proinflammatory Signaling Is Mediated by the NF-***κ***B Pathway

NF-*κ*B is an intracellular protein complex and the central member of a vital and pivotal signaling pathway [54] that plays a key role in immunity [55] and inflammation [56]. Various studies have presented NF-*κ*B as an antiapoptotic and cell cycle control player in malignancies [57–59]. Owing to these qualities, the presence of inflammation and activated NF-*κ*B signaling are risk factors in malignant transformation [60]. The molecular signaling of NF-*κ*B starts with stimulation of receptors for proinflammatory cytokines [56] and certain members of the TNF receptor superfamily, including BAFF-R, TACI, and BCMA [61, 62]. BAFF-R-mediated activation of NF-*κ*B goes through the noncanonical (alternative) signal pathway, whereas TACI and BCMA activate the canonical (classical) NF-*κ*B pathway [8]. NF-*κ*B has the ability to enhance recruitment of inflammatory cells [55] and the expression of proinflammatory cytokines such as IL-1*β* [63, 64], IL-2 [65], IL-6 [66, 67], and TNF-*α* [68]. Deficiency or mutations in the BAFF ligand/receptor system lead to inhibition of NF-*κ*B, thus reducing its antiapoptotic and proinflammatory role [69–71].

## 5. BAFF Is a Biomarker of Disease Progression in Multiple Myeloma

Multiple myeloma (MM) is a malignant disease caused by aberrant proliferation of bone marrow plasma cells. Since BAFF is essential for the survival of B-cells and plays an important role in survival of plasma cells, particularly in early stages of their development, its role in the pathophysiology of multiple myeloma continues to be intensively studied [72, 73]. Serum levels of BAFF in MM patients were found to be significantly higher (6.0 ± 1.88 ng/mL) than in healthy controls (2.25 ± 0.71 ng/mL) in a study by Wang et al. [72] and elsewhere [74–76] and correlated with disease progression and intensity of plasma cell infiltration [76]. Patients with monoclonal gammopathy of unknown significance (MGUS) are reported to have significantly lower serum levels (3.24 ± 0.28 ng/mL) of BAFF and BAFF-R than MM patients [72].

Pretherapeutic, soluble BAFF levels positively correlate with TNF-*α* [72], IL-6 [75, 76], and other adverse markers of disease activity such as C-reactive protein and lactate dehydrogenase in MM patients [75, 76]. Posttreatment levels of BAFF correlate with IL-10, which also modulates apoptosis in B-cells [77], induces proliferation of MM cells [78, 79], and abolishes all-*trans*-retinoic acid inhibitory activity on MM cell growth [79]. Moreover, in the study of Lemancewicz et al., higher serum concentrations of BAFF predicted shorter progression-free survival [75]. Taken together, these clinical studies provide evidence of a strong correlation between BAFF and disease progression in MM.

## 6. BAFF/BAFF-R Signaling May Prove to Be a Promising Target of Future Therapy in B-Cell Derived Malignancies

Simultaneously with MM, the role of BAFF and its receptors was intensively studied in other B-cell derived malignancies such as certain subtypes of non-Hodgkin's lymphomas and precursor B-lineage acute lymphoblastic leukemia (B-ALL). Novak et al. found that BAFF levels corresponded with disease severity and clinical outcome and that elevated levels of BAFF correlated with aggressive phenotype of NHL in humans [80]. Similarly, increased BAFF expression profiles may contribute to* Helicobacter pylori*-independent tumor growth in MALT lymphoma [81]. Elevated levels of BAFF were also reported in other B-lineage lymphomas [82, 83], Hodgkin's lymphoma [84, 85], and B-ALL [82, 86]. Although there is only one FDA-approved anti-BAFF antibody, belimumab, which is used exclusively in rheumatology, new anti-BAFF antibodies are currently being tested for treatment of B-cell lymphomas [87].

In another setting, targeting BAFF-R in B-ALL with a novel humanized anti-BAFF-R antibody selectively kills chemotherapy-resistant precursor B-ALL cells [88]. The anti-BAFF-R antibody also significantly stimulates natural killer cell-mediated killing and macrophage phagocytosis of human ALL cells* in vitro* and decreases leukemia burden in murine bone marrow and spleen. Its therapeutic effects were augmented in combination with conventional chemotherapeutics [89]. BAFF-R might represent a promising therapeutic target because its expression is much higher in leukemic B-cells compared to healthy B-cells [90].

## 7. BAFF Levels Correlate with Disease Activity and Malignant Potential of Cancer Cells in Several Types of Nonhematologic Solid Tumors

Compared to MM and B-derived malignancies, a possible pathophysiological link between BAFF and solid tumors is not as obvious; however, BAFF expression has recently been studied in many types of solid tumors [91–95]. Neuroendocrine tumors (NET) usually express numerous biologically active mediators. Serum levels of BAFF in NET patients (1.195 ± 0.568 ng/mL) are significantly higher compared to healthy controls (0.666 ± 0.240 ng/mL) [94]. Patients in disease progression (1.503 ± 0.637 ng/mL) and patients with metastases (1.391 ± 0.724 ng/mL) have higher serum BAFF levels compared to those with stable disease (0.906 ± 0.273 ng/mL) [94].

BAFF plasma levels were further examined in solid childhood malignancies such as nephroblastoma (Wilms tumor), Ewing sarcoma, and rhabdomyosarcoma showing BAFF levels of 2.757 ± 3.332 ng/mL, 4.311 ± 4.750 ng/mL, and 6.593 ± 4.502 ng/mL, respectively, and these levels were higher compared to the childhood non-Hodgkin's lymphoma subgroup (2.376 ± 1.560 ng/mL) [95].

## 8. BAFF May Contribute to Cancer Cachexia through Its Proinflammatory Properties and by Impairment of the Insulin Receptor Signaling Pathway

Involuntary weight loss is a complication that often follows many serious symptoms such as inanition (inadequate food availability or pathophysiologic conditions substantially decreasing the desire of food), anorexia (reduced food intake caused primarily by diminished appetite with high influence of CNS mechanisms), or cachexia (metabolic disorder of increased energy expenditure leading to a greater weight loss than that caused by reduced food intake alone) [111]. Cancer cachexia is a syndrome where tumors in host organisms play important roles in degrading certain host tissues by production of catabolic mediators [112]. The exact mechanism in which malignant diseases cause cachexia is not completely understood, but there is probably a role for inflammatory cytokines, such as TNF-*α*, various interleukins, and IFN-*γ*, as well as tumor-secreted proteolysis-inducing factor (PIF) and lipolysis mobilizing factor (LMF). Based on these findings, the ghrelin receptor agonist anamorelin hydrochloride has recently been introduced for therapy of cancer-induced cachexia (currently in phase III clinical trials for treatment of cancer cachexia in non-small-cell lung cancer) [113]. Ghrelin binds GHS receptors on T-cells and monocytes and inhibits proinflammatory cytokine expression (IL-1*β*, IL-6, and TNF-*α*). The mechanism of action of anamorelin in cancer cachexia is probably mediated by both a CNS-mediated increase in appetite and anti-inflammatory effects [114]. By inhibition of proinflammatory cytokines and inflammation, anamorelin acts indirectly against BAFF.

**Figure 1:**
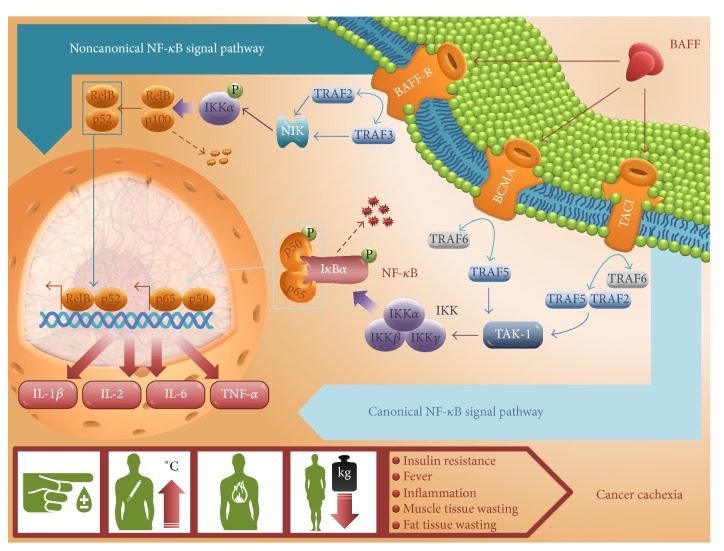
BAFF-induced activation of NF-*κ*B signaling and increased expression of proinflammatory cytokines as procachectic mediators. BAFF interacts with three receptors from the TNF ligand/receptor superfamily, BAFF-R, TACI, and (with lower affinity) BCMA [8]. Upon activation, BCMA signal transduction goes through TNF receptor associated factors (TRAFs) 5 and 6 [96], whereas TACI signals through TRAF2, TRAF5, and TRAF6 [97]. TRAF2 and TRAF5 activate I*κ*B kinase (IKK) via TAK-1 kinase (the canonical NF-*κ*B pathway) [98]. Follow-up phosphorylation of NF-*κ*B inhibitor alpha (I*κ*B*α*) induces ubiquitination of I*κ*B*α* and its proteasome degradation [99]. In this way, I*κ*B*α* is released from the phosphorylated heterodimer p50-p65, and p50-p65 then migrates to the nucleus [99]. BAFF-R signaling starts with TRAF2 and TRAF3 degradation and accumulation of NF-*κ*B inducing kinase (NIK) [100]. In this noncanonical NF-*κ*B pathway, NIK phosphorylates inhibitor of NF-*κ*B kinase alpha (IKK*α*) [101]. IKK*α* then induces cleavage of p100 protein in the p100-RelB complex into a p52-RelB complex which acts as a modulator of nuclear gene transcription [102]. Both canonical and noncanonical NF-*κ*B pathways regulate the expression of genes encoding IL-1*β* [63, 64], IL-2 [65], IL-6 [66, 67], and TNF-*α* [68]. Proinflammatory cytokines participate in manifestation of cancer cachexia symptoms such as insulin resistance [103], fever [104], inflammation [105], and muscle [106–108] and fat tissue wasting [105, 109, 110].

Proinflammatory cytokines target corresponding receptors on host inflammatory and tumor cells and activate the NF-*κ*B signaling pathway [61, 62]. Activation of NF-*κ*B leads to production of even higher amount of cytokines in a positive feedback manner [61, 115]. Binding to its receptors, BAFF enhances NF-*κ*B signaling that leads to increased production of proinflammatory cytokines and promotion of overall inflammation during malignancy (Figure 1) [63–68].

Another common complication arising from the altered metabolism in patients with cancer cachexia is insulin resistance [116]. Hamada et al. found that BAFF-treated mice exhibited increased blood glucose, insulin blood levels, and high expression of TNF-*α*, IL-6, and resistin with decreased expression of adiponectin in visceral adipose tissue, suggesting an impairment in insulin receptor signaling similar to that observed in type II diabetes mellitus and metabolic syndrome. That same study confirmed reduced activation of insulin receptor substrate (IRS-1) as a response to BAFF treatment [40]. BAFF-induced insulin resistance was later confirmed in another mouse model [117]. Insulin resistance augments cancer cachexia in patients with malignancy [112, 118] providing another link between BAFF and the development of cancer cachexia syndrome.

## 9. BAFF Signaling May Contribute to Cancer Progression and Cancer Cachexia Not Just via Its Proinflammatory Role

BAFF may contribute to cancer progression through the amplification of proinflammatory signaling. A causative role of BAFF in cancer and cancer cachexia independent of inflammation has been difficult to substantiate; interestingly however, Koizumi et al. have shown that* in vitro* incubation of tumor cells isolated from pancreatic ductal adenocarcinoma (PDAC) patients with human recombinant BAFF resulted in altered phenotype with increased invasiveness and motility. Downregulation of E-cadherin mRNA and significant upregulation of vimentin and Snail mRNAs were found in these cells. BAFF-induced alteration of epithelial-mesenchymal transition- (EMT-) related genes that support precancerous formations of pancreatic intraepithelial neoplasias and PDAC itself was confirmed on BAFF-R overexpressing cell clones [91]. Thus, BAFF may promote tumorigenesis indirectly by induction of inflammation in the tumor microenvironment and directly by induction of EMT.

Similar to BAFF's involvement in cancer progression, BAFF's involvement in cancer cachexia is difficult to distinguish from its proinflammatory effects. BAFF may contribute to cancer cachexia by affecting changes in NF-*κ*B pathway-induced inflammation and through impairment of insulin sensitivity via reduction of adiponectin and possibly other adipokines maintaining glucose homeostasis.

**Figure 2:**
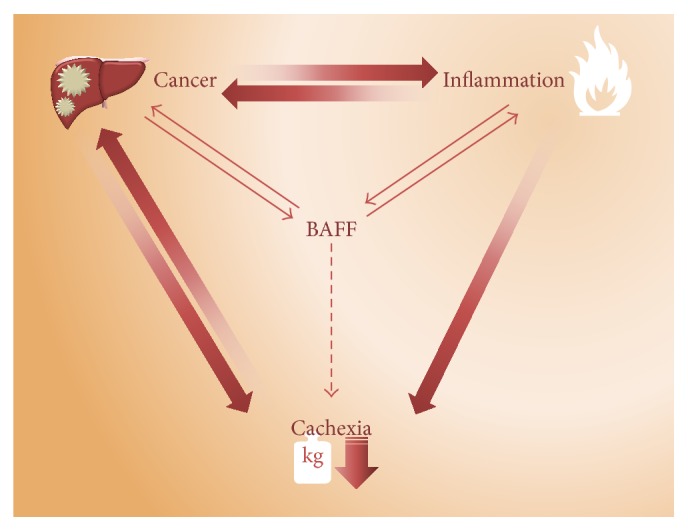
BAFF in cancer cachexia interplay. Outer arrows indicate well-described hallmarks of cancer cachexia. Cancer → inflammation: many types of cancer cells express cytokines that induce inflammation [119]. Inflammation → cancer: tumors often manifest on inflammatory background that supports transition of cells to malignant clones (e.g., hepatocellular carcinoma or PDAC as cited in the text). Cancer → cachexia: tumor tissue directly participates in the development of cancer cachexia by production of tumor specific factors like PIF and LMF [120, 121]. Cachexia → cancer: cachexia in cancer patients remains a significant cause of morbidity and mortality in cancer treatment [122]. Inflammation → cachexia: proinflammatory cytokines induce cachexia by increased catabolism with altered insulin sensitivity [119]. Inner arrows indicate established (solid line) and putative (dashed line) role of BAFF in pathophysiology of cancer cachexia. Cancer → BAFF: increased expression and serum levels of BAFF were demonstrated in many types of hematological and solid tumors making BAFF a possible new biomarker in malignancies. BAFF → cancer: BAFF has been found to augment manifestation of lymphoma and the formation of epithelial-mesenchymal transitions and pancreatic intraepithelial neoplasias. These events precede PDAC. (1) A TNF-independent role of BAFF in the pathophysiology of lymphomas was demonstrated in BAFF-Tg TNF^−/−^ mice. More than 35% of BAFF-Tg TNF^−/−^ mice had occurrence of various types of lymphomas within 1 year [123]. (2) BAFF-induced alteration of the epithelial-mesenchymal transition- (EMT-) related genes that support precancerous formation of pancreatic intraepithelial neoplasias and PDAC was confirmed on BAFF-R overexpressing cell clones [91]. Inflammation → BAFF: BAFF is produced by several proinflammatory cells. BAFF → inflammation: BAFF induces expression of proinflammatory cytokines by activation of NF-*κ*B [124]. BAFF → cachexia: BAFF induces insulin resistance [40, 117] which has been associated with cancer cachexia [116, 118].

Taken together, an increase in catabolic demands during inflammation and malignancy predispose to cancer cachexia development. BAFF may enhance the inflammatory background in cancer patients, providing a tantalizing link to involvement in cancer cachexia (Figure 2); however additional studies will be required to confirm such a link and potential avenue for therapeutic intervention.

## Figures and Tables

**Table 1:** Overview of BAFF receptors.

	Gene	Forms	Ligands	Affinity to BAFF (*K*_*D*_)	Tissue expression	Function	Clinical relevance
BAFF-R (BAFF-receptor, TNFRSF13C, BLyS, BR3, CD268) [13]	22q13.2 3 exons [13]	Membrane bound, soluble (produced by decidual cells) [8, 14]	BAFF [8]	16 nmol·l^−1^ [8]	B-, T-cells [15, 16] mature and immature adipose tissue [17]	B-cell proliferation [18], T-cell proliferation [16]	BAFF-R is constitutively saturated in autoimmune and lymphoproliferative diseases [15, 19, 20]

TACI (transmembrane activator and calcium signal-modulating cyclophilin ligand, TNFRSF13B, CD267) [21]	17p11.2 5 exons [21]	Membrane bound [8]	BAFF, APRIL [8]	146 nmol·l^−1^[22]	B-, T-cells immature adipose tissue [17]	T-cell activation [23] and humoral immunity response modulation [24, 25]	Mutations may result in common variable immunodeficiency [26, 27]

BCMA (B-cell maturation antigen, TNFRSF17) [28]	16p13.1 3 exons [28]	Membrane bound [8]	APRIL (BAFF) [8]	1600 nmol·l^−1^ [8]	B-cells immature [17]	Long-term plasma cell survival, B-cell antigen presentation [29]	Protection of multiple myeloma cells from apoptosis [30, 31]
